# Loss of a 20S Proteasome Activator in *Saccharomyces cerevisiae* Downregulates Genes Important for Genomic Integrity, Increases DNA Damage, and Selectively Sensitizes Cells to Agents With Diverse Mechanisms of Action

**DOI:** 10.1534/g3.112.003376

**Published:** 2012-08-01

**Authors:** Kevin M. Doherty, Leah D. Pride, James Lukose, Brian E. Snydsman, Ronald Charles, Ajay Pramanik, Eric G. Muller, David Botstein, Carol Wood Moore

**Affiliations:** * Department of Microbiology and Immunology, City University of New York Sophie Davis School of Biomedical Education, City College, New York, New York 10031-9101; † The Graduate Center Program in Biochemistry, City University of New York, New York, New York 10016-4309; ‡ Department of Biochemistry, City College, City University of New York, New York, New York 10031-9101; § Department of Biochemistry, University of Washington, Seattle, Washington 98195-7350; ** Lewis-Sigler Institute for Integrative Genomics and Department of Molecular Biology, Princeton University, Princeton, New Jersey 08544-1004, and; †† Graduate Center Programs in Biochemistry and Biology, City University of New York, New York, New York 10016-4309

**Keywords:** 20S proteasome activator, *BLM10*/PA200, *UBP3*/*BLM3*, DNA damage, molecular chaperones

## Abstract

Cytoprotective functions of a 20S proteasome activator were investigated. *Saccharomyces cerevisiae* Blm10 and human 20S proteasome activator 200 (PA200) are homologs. Comparative genome-wide analyses of untreated diploid cells lacking Blm10 and growing at steady state at defined growth rates revealed downregulation of numerous genes required for accurate chromosome structure, assembly and repair, and upregulation of a specific subset of genes encoding protein-folding chaperones. Blm10 loss or truncation of the Ubp3/Blm3 deubiquitinating enzyme caused massive chromosomal damage and cell death in homozygous diploids after phleomycin treatments, indicating that Blm10 and Ubp3/Blm3 function to stabilize the genome and protect against cell death. Diploids lacking Blm10 also were sensitized to doxorubicin, hydroxyurea, 5-fluorouracil, rapamycin, hydrogen peroxide, methyl methanesulfonate, and calcofluor. Fluorescently tagged Blm10 localized in nuclei, with enhanced fluorescence after DNA replication. After DNA damage that caused a classic G2/M arrest, fluorescence remained diffuse, with evidence of nuclear fragmentation in some cells. Protective functions of Blm10 did not require the carboxyl-terminal region that makes close contact with 20S proteasomes, indicating that protection does not require this contact or the truncated Blm10 can interact with the proteasome apart from this region. Without its carboxyl-terminus, Blm10_(−339aa)_ localized to nuclei in untreated, nonproliferating (G_0_) cells, but not during G_1_ S, G_2_, and M. The results indicate Blm10 functions in protective mechanisms that include the machinery that assures proper assembly of chromosomes. These essential guardian functions have implications for ubiquitin-independent targeting in anticancer therapy. Targeting Blm10/PA200 together with one or more of the upregulated chaperones or a conventional treatment could be efficacious.

Aggregated, unfolded, misfolded, and nonfunctional proteins accumulate in many human diseases, such as cancers (Coughlan and Brodsky 2003; Scott and Frydman 2003; Huo 2010; Nagaraj *et al.* 2010). Proteasomes degrade such proteins, along with those otherwise damaged or altered or no longer needed (Dobson 2003; Goldberg 2003; Demartino and Gillette 2007; Rosenzweig and Glickman 2008). These multicatalytic proteinase complexes conduct the preponderance of intracellular protein degradation and dispense with DNA-damaging agents or other toxic compounds in cells. Selective inhibition of proteasomes in cancer cells is an anticancer treatment strategy whose efficacy lies in blocking metabolic functions, inducing apoptosis, and sensitizing malignant cells and tumors to chemotherapeutic agents and radiation (Voorhees and Orlowski 2006; Orlowski and Kuhn 2008; Yang *et al.* 2009).

Proteolytic activities of proteasomes occur inside 20S multisubunit core particles, of which homologous human proteasome activator 200 (PA200) and yeast Blm10 are activators and regulatory proteins (Schmidt *et al.* 2005b; Főrster and Hill 2006; Finley 2009; Stadtmueller and Hill 2011; Savulescu and Glickman 2011; Lopez *et al.* 2011; Dange *et al.* 2011). We found PA200 widely distributed in adult human tissues but not fetal tissues (Febres *et al.* 2001), and others found it widely distributed in mouse tissues (Ustrell *et al.* 2002) and required for normal spermatogenesis (Khor *et al.* 2006). The yeast and human proteins share 17% sequence identity (Ustrell *et al.* 2002; Ortega *et al.* 2005; Iwanczyk *et al.* 2006). It was actually the divergent sequences that led to the prediction that PA200 and Blm10 may perform different *in vivo* roles (Főrster and Hill 2006).

Blm10 was first discovered as a multicopy suppressor (Febres *et al.* 2001; Doherty *et al.* 2004) of the hypersusceptibilities to killing by anticancer bleomycins and structurally related phleomycins that are conferred by the *blm3-1* mutation (Moore 1991). This nonsense mutation in the *UBP3/BLM3* (human *Ubp10/Usp10*) ubiquitin-specific protease gene truncates upstream of the ubiquitin hydrolase domain (McCullock *et al.* 2006) (Figure 1). In addition to bleomycin and phleomycin (Moore 1991; Febres *et al.* 2001; McCullock *et al.* 2006), the *blm3-1* mutation confers hypersusceptibilities to lethal effects of gamma irradiation and hydrogen peroxide (Moore 1991) and canavanine, hydroxyurea, and growth at 37° (McCullock *et al.* 2006). It was proposed that Ubp3 promotes protein stability by deubiquitinating misfolded proteins, permitting their refolding and function (Brew and Huffaker 2002). Genetic interaction data suggest a role for Ubp3 in transcriptional elongation (McCullock *et al.* 2006). It was suggested that Ubp3 physically interacts with the 26S proteasome and the Rad4 protein to facilitate degradation of Rad4 and suppression of DNA nucleotide excision repair (Mao and Smerdon 2010).

**Figure 1:**
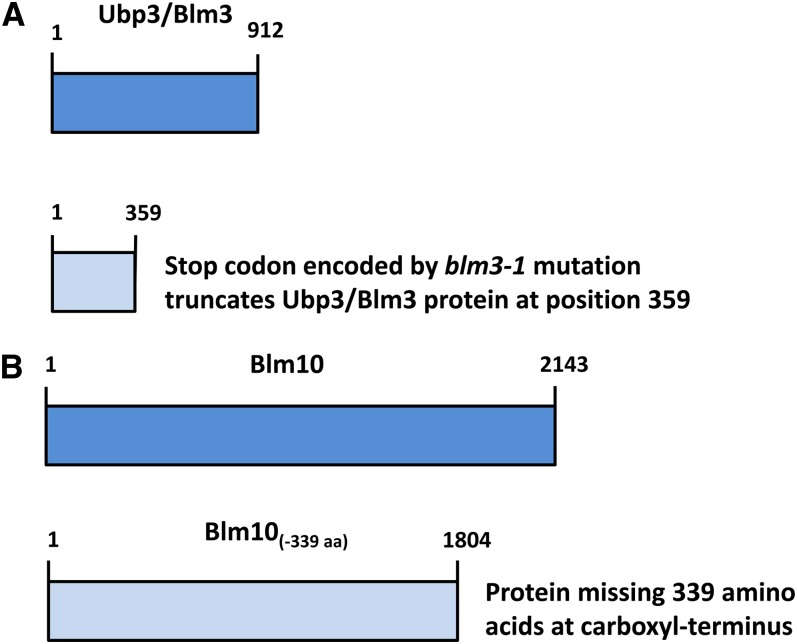
Truncations of the Blm10 and Ubp3/Blm3 proteins as described in the text. Dark blue indicates full-length proteins; light blue: truncated proteins.

As energy-independent 20S proteasome activators, Blm10 and PA200 do not require ATPases and ubiquitinated substrates for activation (Ustrell *et al.* 2002; Schmidt *et al.* 2005a). Structural and biochemical properties of Blm10/PA200 were recently reviewed (Stadtmueller and Hill 2011; Savulescu and Glickman 2011; Lopez *et al.* 2011; Dange *et al.* 2011). Electron microscopy (Schmidt *et al.* 2005a; Iwanczyk *et al.* 2006) and crystal structure (Sadre-Bazzaz *et al.* 2010) show Blm10 docks onto the axial end of the core particle cylinder, allowing it to regulate the state of the core particle channel. Active gate opening by Blm10 engages its carboxyl-terminus with the core particle (Dange *et al.* 2011). In proteasome assembly and maturation, Blm10 associates with nascent and *de novo* synthesized 20S core particles (Fehlker *et al.* 2003); caps the core particle in its association with stable, mature complexes (Schmidt *et al.* 2005a); and binds to preactivated core particles (Lehmann *et al.* 2008). The protein is detected in association with mature proteasomes (Schmidt *et al.* 2005a; Iwanczyk *et al.* 2006), and half (Li *et al.* 2007; Marques *et al.* 2007) and full (Fehlker *et al.* 2003; Li *et al.* 2007; Marques *et al.* 2007) precursor complexes. Although initial computer modeling of the predicted Blm10 amino acid sequence led to its classification as a potential membrane transport protein containing seven to 10 transmembrane domains (Febres *et al.* 2001), these are now known to be HEAT domains (Kajava *et al.* 2004). HEAT repeat proteins have a characterized solenoid structure that facilitates Blm10 binding to the core particle surface, wrapping around the core particle, and looping into the catalytic chamber to interact with core components. PA200 attaches to the α-ring surface in a defined conformation, coming into contact with all subunits except α7 (Glickman and Raveh 2005; Ortega *et al.* 2005).

Although strains with the *BLM10* gene deleted are hypersusceptible to the lethal effects of bleomycin and phleomycin (Febres *et al.* 2001; Doherty *et al.* 2004; Schmidt *et al.* 2005a), no evidence exists that Blm10 or PA200 performs a direct role in DNA repair. PA200 was previously reported to be involved in DNA repair based on the change of finely punctated patterns of PA200 in HeLa nuclei to foci after gamma irradiation but not after hydrogen peroxide or ultraviolet light treatments (Ustrell *et al.* 2002). It is now known that this form of cellular PA200 is found associated with proteasomes and that PA200 in association with proteasomes, rather than independently, accumulates on chromatin after ionizing irradiation (Blickwedehl *et al.* 2007). Consistent with these findings, it is known that proteasomes in yeast associate with sites of DNA double-strand breaks (Krogan *et al.* 2004).

The purpose of the current studies was to investigate some of the properties conferred by the *BLM10* gene. Because of the valuable biology that can be obtained from the comprehensive and simultaneous analyses of thousands of genes, we used the technologies of systems biology to measure and compare global gene expression in cells with and without Blm10. To obtain this systems-level understanding, we sought to identify differentially coexpressed genes and discover some of the interactive networks and pathways affected by the loss of the activator irrespective of whether the regulated genes may be directly or indirectly due to the absence of Blm10. We grew strains under steady-state growth conditions in chemostats to avoid confounding changes in gene expression caused by changes in growth rates between normal and mutant strains (Brauer *et al.* 2008). We followed up the comparative genomic analyses with comparative studies of global chromosomal integrity. We investigated a potential role for Blm10 or Ubp3/Blm3 in maintaining chromosomal integrity after DNA damage and a potential role of Blm10 in protecting against agents with different mechanisms of action. In protein localization experiments, we constructed a YFP-Blm10 fusion protein and used it to track Blm10 localization during the cell cycle and before and after DNA damage. Functions of the Blm10 carboxyl-terminus in protection and proper localization were also examined. Blm10 activation or regulation of the 20S proteasome is shown to be an important step in properly assembling chromosomes. The findings provide important new insights into the molecular mechanisms of protection and genomic stability conferred by Blm10 and suggest Blm10/PA200 inhibition could hold promise as a novel approach to cancer treatment.

## Materials and Methods

### Strains, plasmids, and primers

Yeast strains, plasmids, and primers used in these studies are listed, respectively, in Table 1, Table 2, and Table 3. Transformations and plasmid isolations were adapted from published methods (Rose and Broach 1991; Febres *et al.* 2001; Gietz and Woods 2002). For chemostat experiments, pulsed-field gel electrophoretic analyses, survival, and susceptibility experiments, we used diploid strains to avoid suppressors that could mask or modify mutant phenotypes.

**Table 1:** Yeast strains

Strain	Genotype	Source
BESY54	derived from M1452-98B. *ade2-40 ilv1-92 trp1-1 ura3-1* YFP-*BLM10 SPC42*-CFP	Eric Muller
BMA-8A	*MAT***a** *ura3-52 trp1Δ63 leu2Δ his3Δ200 gal2*	Agnes Baudin
CM1452-98B	*MAT*α *ura3-52 ade2-40 leu2-3 ilv1-92*	This laboratory
CM1469-5A	*MAT*α *ilv1-92 his3-11* or *his3-15 leu2-3*, *112 trp1-1 ura3-1*	This laboratory
CM1469-5C	*MAT***a** *ade2-40* or *ade2-1 ura3-1 ilvl-92 trp5-12 ura3-1 blm3-1*	This laboratory
CM1522-9B	*MAT***a** *ura3-52 trp1Δ63 leu2Δ his3Δ200 blm10Δ*::*HIS3*	This laboratory
CM-1526	*MAT***a***/MAT*α *ade2-40/ade2-40 trp1-1/trp1-1 HIS3/his3-11 LEU2/leu2-3 ura3-1/ura3-1 ILV1/ilv1-92 blm3-1/blm3-1*	This laboratory
CM-1527	*MAT***a***/MAT*α *LEU2/leu2-3 ILV1/ilv1-92 HIS3/his3-11 ade2-40/ade2-40 TRP1/trp1-1*	This laboratory
CM-1528	*MAT***a***/MAT*α *LEU2/leu2-3 ade2-40/ade2-40 trp1-1/trp1-1 URA3/ura3-1 ILV1/ilv1-92 BLM3/blm3-1*	This laboratory
CM-1529	*MAT***a***/MAT*α *LEU2/leu2-3 URA3/ura3-1 ILV1/ilv1-92 ade2-40/ade2-40 trp1-1/trp1-1*	This laboratory
CM1530-1A	*MAT***a** *ura3-52 trp1Δ63 leu2Δ his3Δ200 gal2 blm10Δ*::*HIS3*	This laboratory
CM-1531	*MAT***a***/MAT*α *trp1/trp1 leu2/leu2 ura3/ura3 blm10Δ*::*HIS3/ blm10Δ*::*HIS3*	This laboratory
CM1531-1B	*MAT*α *ura3-52 ade2-40 leu2-3 ilv1-92 BLM10*::*YFP*	This laboratory
DBY9500 (CEN.PK)	*MAT***a***/MAT*α *MAL2-8C/ MAL2-8C*	Maitreya Dunham
EJ758	*MAT***a** *his3-D200 leu2-3,112 ura3-52 pep4*::*URA3*	Eric Phizicky, Elizabeth Jones

**Table 2:** Plasmids

Plasmid Name	Experiment	Parental Vector	*Escherichia coli* Selection	Yeast Selection	Tag	Insert
pRS303	Deletion	pBluescript	Amp	*HIS3*	None	*HIS3*
pDH22	Localization		Amp	*Kan^r^*	None	Kan and YFP
pSH47	Localization		Amp	*URA3*	None	Cre recombinase
pUB23	β-galactosidase			*URA3*		
pYEX 4T-1B	GST	pYEULC	Amp	*URA3*	GST	*BLM10*

GST, Glutathione-*S*-transferase.

**Table 3:** Oligonucleotide primers

Primer Name	Primer Use	Length (Bases)	Sequence 5′-3′
1	Sequencing	20	ATATGCCGCAGACGGAAGAC
2	Sequencing	20	ATATAAGACTGAAAGTCATG
3	Sequencing	21	GCCTATCGTTACATCCGTTGT
4	Sequencing	21	AGTAATTCGGTTTATTGTGAT
5	Sequencing	19	CAAAGAACAAATCAAAAGA
6	Sequencing	20	TCAGTGGCACGTACCTTCTA
7	Sequencing	21	CTTCATTGACGTTGATTTCCT
8	Sequencing	22	CAAAAAGAAAAAGCGTGAGTAC
9	Sequencing	22	AAAGCTCAATTTACGTGAGAAT
10	Sequencing	22	GTTGGTATTTGATCACCCATAC
11	Sequencing	21	GTTCGTGCGGCATCCATTTTG
PA-05	Sequencing/deletion verification/YFP verification	30	GCGCGGTACCATTACGCAGAATAATCTATG
YFP-up	YFP cassette	94	TTCAATTGGGATAAGGTCTTGTTAGTAATGGGAAT
			GGGTGATTTGATATCATCGTCATTGTTAGCGGTCAT
			TTTGTACAATTCATCCATACCATG
YFP-down	YFP cassette	82	TTGCATACATAAACTTTATCATTGTTCGTTAGCTAG
			CTTTGCACATTAATTTTTCGATTTGTTACCGCCACGG
			CCGCCAGGG
Deletion primer 1	Replacement cassette	72	ATGATCTCAAACTGCTTCTTAATATAGGCATCCAC
			CTTTTCTGGGACGCTTTTTACTCTTGGCCTCCTCTAG
Deletion primer 2	Replacement cassette	68	CAAATCTACATGTATATACAGATCTATACAGCAA
			TTATAGGATATCTTTCGTTCAGAATGACACG
HIS3 R	Verify deletion	21	CAGACAATCAACGTGGAGGGT
NF	Sequencing	22	ATTCCCATTACTAACAAGACCT
NR	Sequencing	22	ATCGCAATATAAAGATTAACTA
L1F	Sequencing	22	AATCTTATATTGCGATCAGCTC
L1R	Sequencing	24	GATATGATAAGATAGGGCACAAC
L2F	Sequencing	24	GGGATTTTTACTGATGATCAAATG
L2R	Sequencing	25	GATATGATAATGATAGGGCACAAC
L3F	Sequencing	24	TGTTTAACTTCTTTTTGTCACGAA
L3R	Sequencing	25	GATAGGAATGAAAGCGGCTATAGA
L4F	Sequencing	24	AACCTCATCAACGGTATTGTATCT
L4R	Sequencing	24	TATTTCGGTTGTACATAGAGTTGC
L5F	Sequencing	25	ACTCTATGTACAACCGAAATAACTG
L5R	Sequencing	21	AAATATCAATCTGCCGATGTC
L6F	Sequencing	25	AGTGTATGTGTCATTTCCGATCAAG
L6R	Sequencing	23	CATATTCAGTTCGCAGAAACCAG
CF	Sequencing	24	TCATCTGGTTTCTGCGAACTGAAT
CR	Sequencing	25	GTTAGCGACAGCTGGCGAACCTGA

### Deletion of *BLM10*

Using polymerase chain reaction (PCR) deletion and replacement methods (Baudin *et al.* 1993), chromosomal *BLM10* was deleted in haploid strains by PCR amplification of the *HIS3* replacement cassette in plasmid pRS303 (kindly provided by Dr. Susan Henry), transformation of the PCR product into strain BMA-8A (kindly donated by Dr. Agnes Baudin), selection of *HIS3*^+^ recombinants, and verification by PCR of the correct gene deletion using oligonucleotides complementary to *BLM10* flanking sequences (Table 3). Segregants from heterozygous diploids sporulated in 0.25% yeast extract, 0.1% glucose, 0.98% potassium acetate, and 40 mg/mL uracil, tryptophan, and leucine were intercrossed.

### Chemostats

Detailed procedures for chemostat setup, temperature and pH probes, glucose-limited medium, aeration, daily monitoring, data acquisition, harvests, sample processing, and RNA preparations were followed as described at http://dunham.gs.washington.edu/protocols.shtml. Chemostats with individually calibrated pH and temperature probes were established in 500-mL fermenter vessels (Sixfors; Infors AG, Bottmingen, Switzerland) containing 300-mL cultures. They were stirred at 400 rpm with 5 L per minute of humidified and filtered air. Equivalent population growth (dilution) rates and doubling times were confirmed by measuring effluent volumes over time, microscopic examination, and cell counts.

### Microarrays

RNA was isolated, labeled, and hybridized according to the instructions supplied by Agilent. Reference RNA was isolated from diploid strain DBY9500 (CEN.PK; *Mal2-8C/Mal2-8C*), labeled, and used in all hybridization samples. Cells had been grown to steady state in a chemostat and kindly provided by Dr. Maitreya Dunham.

### Analyses of gene expression data

Mean expression values were calculated among replicated experiments and microarrays. Data were analyzed using the Agilent Scanner and Feature Extraction software, Princeton University MicroArray database (Gollub *et al.* 2003), Gene Ontology Local Exploration Map (GOLEM) software (Sealfon *et al.* 2006), *Saccharomyces* Genome Database (SGD 2010), and bioPIXIE (biological Process Inference from eXperimental Interaction Evidence) software (Myers *et al.* 2005). Results are presented for dilution rate = 0.06 hr^−1^ (doubling time = ∼12 hr) and were comparable for dilution rate = 0.12 hr^−1^ (doubling time = ∼6 hr).

### β-galactosidase assays

Strains CM1469-5A (*BLM10*, *UBP3/BLM3*), CM1522-9B (*blm10*Δ, *UBP3/BLM3*), and CM1469-5C (*BLM10*, *blm3-1*) are auxotrophic for uracil. They were grown in synthetic selective media lacking uracil to select the *URA3* gene on plasmid pUB23 (kindly provided by Dr. Alfred Goldberg). After induction of the fusion protein by galactose, β-galactosidase was assayed as published (Rose and Botstein 1983).

### Pulsed-field gel electrophoreses and survival

Pulsed-field gel electrophoresis and cell survival were measured in parallel in each experiment as previously described (Moore *et al.* 2000). Phleomycin was supplied and prepared as previously described (Moore 1982, 1988). Concentrations were determined using the Beer-Lambert equation (OD_245_ /1.6 × 10^−2^). Liquid-holding (LH) recovery was measured under nondividing, non-replicating conditions maintaining full viability (Moore *et al.* 2000).

### Susceptibility tests

Fresh cells were harvested from overnight cultures grown in standard, nonsynthetic complete medium (YPAD; Moore 1982) with aeration at 30° to 5 × 10^8^ cells/mL, washed twice with deionized water to remove growth medium, and resuspended at 5 × 10^7^ cells/mL in deionized water. Five microliters of several dilutions were pipetted on YPAD plates, prepared the preceding day, with varying dilutions of 1% methyl methanesulfonate (Sigma-Aldrich), hydrogen peroxide (3% stabilized, pharmaceutical grade), 0.02 M 5-fluorouracil (3 mg/mL; City Chemical), 5 M hydroxyurea (0.38 g/mL; Sigma-Aldrich), 0.001 M doxorubicin chlorhydrate (adriamycin, Tecoland; 0.58 mg/mL DMSO [Fisher Scientific]), rapamycin/sirolimus (Tecoland; 0.09 mg/mL), or calcofluor white (American Cyanamid). Plates were incubated at 30° for up to 4 days.

### Yellow fluorescent protein (YFP) fusion and fluorescence microscopy

YFP was fused to the N-terminus of Blm10 as previously described (Prein *et al.* 2000). The fusion complements a Blm10 deletion mutant as judged by its total relief of hypersensitivity to phleomycin. CM1452-98B (YFP-Blm10) and BESY54 (YFP-Blm10, Spc42-CFP) were grown on nonsynthetic complete medium and imaged using a DeltaVision microscopy system from Applied Precision (Issaquah, WA). The system incorporates an Olympus IL-70 microscope, a u-plan-apo 100× oil objective (1.35NA), a CoolSnap HQ digital camera from Roper Scientific (Tucson, AZ), and optical filter sets from Omega Optical (Brattleboro, VT). Live cells were imaged on thin pads of medium containing 1% agarose (Muller *et al.* 2005). Images were analyzed and quantitated using the program Fluorcal, an integrated set of Matlab scripts designed for the automated selection and analysis of regions of interest within images obtained by fluorescence microscopy (Shimogawa *et al.* 2010). Phleomycin D_1_ was purchased from Invitrogen (Life Technologies, Carlsbad, CA) in the formulation of Zeocin.

### Glutathione-*S*-transferase (GST) fusion, immunofluorescence, and quantitative expression of Blm10_(-339aa)_

Truncated Blm10-GST under control of the *CUP1* promoter was purified from a pool of slightly modified plasmid pYEX 4T-1 (kindly provided by Dr. Eric Phizicky; Martzen *et al.* 1999), confirmed by DNA sequencing, and transformed into *blm10Δ* cells. To localize Blm10-GST, cells were grown to early exponential phase (10^6^−10^7^ cells/mL) in liquid YPAD containing copper (50 μM) to induce expression of fusion protein. Cells were fixed by adding 3.7% formaldehyde directly to the medium, incubated for at least 1 hr, converted to spheroplasts in 1 mL of 50 μL/mL Zymolyase 100T in 0.1 M potassium phosphate (pH 7.5) with 2 μL/mL 2-mercaptoethanol, gently pelleted, and treated with Alexa Fluor 488 anti-GST conjugate antibody (Molecular Probes) or 4′,6-diamidino-2-phenylindole (DAPI, 1 mg/mL in water). Cells were observed using a Zeiss fluorescent microscope at 100× with FITC and DAPI filters.

To quantitate growth and survival, copper and phleomycin were added to fresh cells grown overnight in YPAD, washed, and resuspended in YPAD at 5 × 10^6^ cells/mL. Cells were counted and plated on YPAD at each time point to determine viability.

## Results

### Genomic instability signature of downregulated genes

To determine how cells alter their gene expression in the absence of the Blm10 protein, we studied microarray-based global gene expression for the entire genome (∼6000 genes) in normal (*BLM10/BLM10*) and mutant (*blm10Δ/ blm10Δ*) diploids in the context-specific environments of chemostats. Diploids of both genotypes were grown in continuous steady-state at the same controlled growth rates (Brauer *et al.* 2005; Kubitschek 1970). In these steady-state growth conditions, the growth rate is determined by the medium flow rate and therefore identical between normal and mutant cultures. This was an important part of the experimental design because we found *blm10Δ/blm10Δ* cells grow slowly outside of chemostats in some media in comparison with *BLM10Δ/BLM10Δ* cells of the same genetic background.

To our surprise, many genes specifically encoding proteins required for proper chromosome organization, assembly, function, repair, and progression through the cell cycle were downregulated to different extents in *blm10Δ/blm10Δ* cells. Some of these are shown in Table 4, along with their human homologs. Among these were genes encoding 18 DNA packaging, nucleosome organization, and chromatin assembly proteins; nine DNA damage and checkpoint proteins; seven DNA repair proteins; eight transcription factors; and 34 proteins assuring correct numbers of chromosomes segregate during cell division. Twenty-five additional genes involved in nucleosome modeling and DNA packaging were downregulated more than 25% (supporting information, Table S1). In addition, the recombinational repair gene, *RAD51*, was downregulated 62%, and all six remaining *YRF1* helicase genes were downregulated 76–91% (Table S1).

**Table 4:** Some of the significantly downregulated genes critical for chromosomal integrity

	Genes
Chromosome organization and function	
DNA packaging, nucleosome organization, chromatin assembly or dissassembly, nucleosome and chromatin remodeling, chromatin organization and modification	***ACS2*** 2.8, *HST3* 2.3, ***HHF1*** 2.2, ***HTZ1*** 2.2, ***HHO1*** 2.1, ***NHP6A*** 2.1, ***HTB1*** 2.0, ***HTB2*** 2.0
DNA repair: double-strand break repair, mismatch repair, postreplication repair	*RAD59* 2.4, *MCK1* 2.3, *NHP6A* 2.1, ***HTB1*** 2.0, *PSY3* 1.8, ***MEC3*** 1.6, ***DUN1*** 1.5
Mitotic DNA recombination, telomere arrangement and maintenance	*RAD59* 2.4, ***HHO1*** 2.1, *YRF1*-*3* 2.0, *YRF1*-*6* 2.0, *PSY3 1.8*, ***MEC3*** 1.6
Regulation and progression of cell cycle, cyclin-dependent protein kinase activity	***CLB2*** 3.3, *PCL9* 3.2, ***SIC1*** 2.6, ***CLB1*** 2.0, ***MEC3*** 1.6
G1/S transition of mitotic cell cycle	*PCL9* 3.2, ***SIC1*** 2.6
Mitotic spindle assembly and chromosome segregation	*CIN8* 2.4
Spindle pole body separation, microtubule cytoskeleton organization, G2/M transition	***CLB2*** 3.3, ***CLB1*** 2.0, ***HSL1*** 1.9
Chromatin silencing and negative regulation of gene expression, epigenetic	***HTZ1*** 2.2, ***MEC3*** 1.6
Meiotic DNA replication, meiotic DNA recombination	*RIM4* 2.2, *MUM2* 2.1
Checkpoints	
Genome integrity, DNA damage sensor	***MEC1*** 1.6, ***MEC3*** 1.6, ***SGS1*** 1.6, ***DUN1*** 1.5
DNA replication, gap repair of damaged DNA	*TOF1* 1.5
Septin	***HSL1*** 1.9, *GIN4* 1.7
Spindle checkpoint activation, protects sister chromatid cohesion in mitosis	***PHB2*** 1.6
Meiotic recombination	***FPR3*** 1.7

Cytokinesis	
Cytokinesis, cell division	*DSE2* 5.4, *SUN4* 5.3, *DSE1* 4.6, *CTS1* 4.1, *SCW11* 3.7, *CHS2* 3.5, *BUD9* 3.2, *EGT2* 2.8, *HOF1* 2.5, ***MYO1*** 2.3, *RAX1* 2.3, ***IQG1*** 2.2, ***HSL1*** 1.9
Transcription factors	
G1 cell-cycle progression, cyclin-dependant kinase target	*SFG1* 3.6
Activates expression of early G1-specific genes	*ACE2* 2.6
Activates transcription of genes expressed at M/G1 and G1, activates cyclin Pcl9	*SWI5* 2.5
Response to DNA damage stimulus, expression highest in G1	*TOS4* 1.8
Response to DNA damage stimulus, progression from G1 to S and G2 to M	***CKA2*** 1.6
Involved in directing transcription of genes by RNA polymerases, I, II, and III	***SPT15*** 1.5
RNA polymerase II initiation and elongation	***TFG2*** 1.5
Calcineurin B; calcineurin regulates stress-response transcription factor Crz1; human protein participates in apoptosis and other signaling pathways	***CNB1*** 1.5

Genes are listed in each subgroup or group in order of their fold decreased expression. Essential genes are underlined. Yeast genes in bold have the following human homologs: *ACS* (***ACS2***), *ATR* (***MEC1***), *BLM* and *WRN* (***SGS1***), *BRSK2* (***HSL1***), *CCNB1* (***CLB1***), *CCNB2* (***CLB2***), *CHEK2* (***DUN1***), *CSNK2A1* (***CKA2***), *FPR3* (***FPR3***), GSK3 family (***MCK1***), *H1F0* (***HHO1***), *H2AFV*, (***HTZ1***), *HIST1H2BH* (***HTB1***), *HIST1H2BO* (***HTB2***), HIST1H4N (***HHF1***), *HMGB1/HMG1* (***NHP6A***), *Hus1* (***MEC3***), *IQGAP1* (***IQG1***), *Kip1* (***SIC1***), and *MYH11* (***MYO1***), *PHB2* (***PHB2***), *PPP3R2* (***CNB1***), *RAP30* (***TFG2***), *TBP* (***SPT15***).

While striking, the downregulation of all of these genes may not necessarily decrease protein expression in all instances after posttranscriptional and posttranslational modification, or actually be a direct rather than indirect consequence in all cases by the absence of Blm10. Moreover, the actual levels of downregulation may not be strictly quantitative. However, what is most important and remarkable is the unique and composite signature defined by the simultaneous downregulation of the genes. As a whole, the results suggest a vulnerability of cells without Blm10.

### Chaperone-mediated, protein-folding signature of upregulated genes

**Figure 2:**
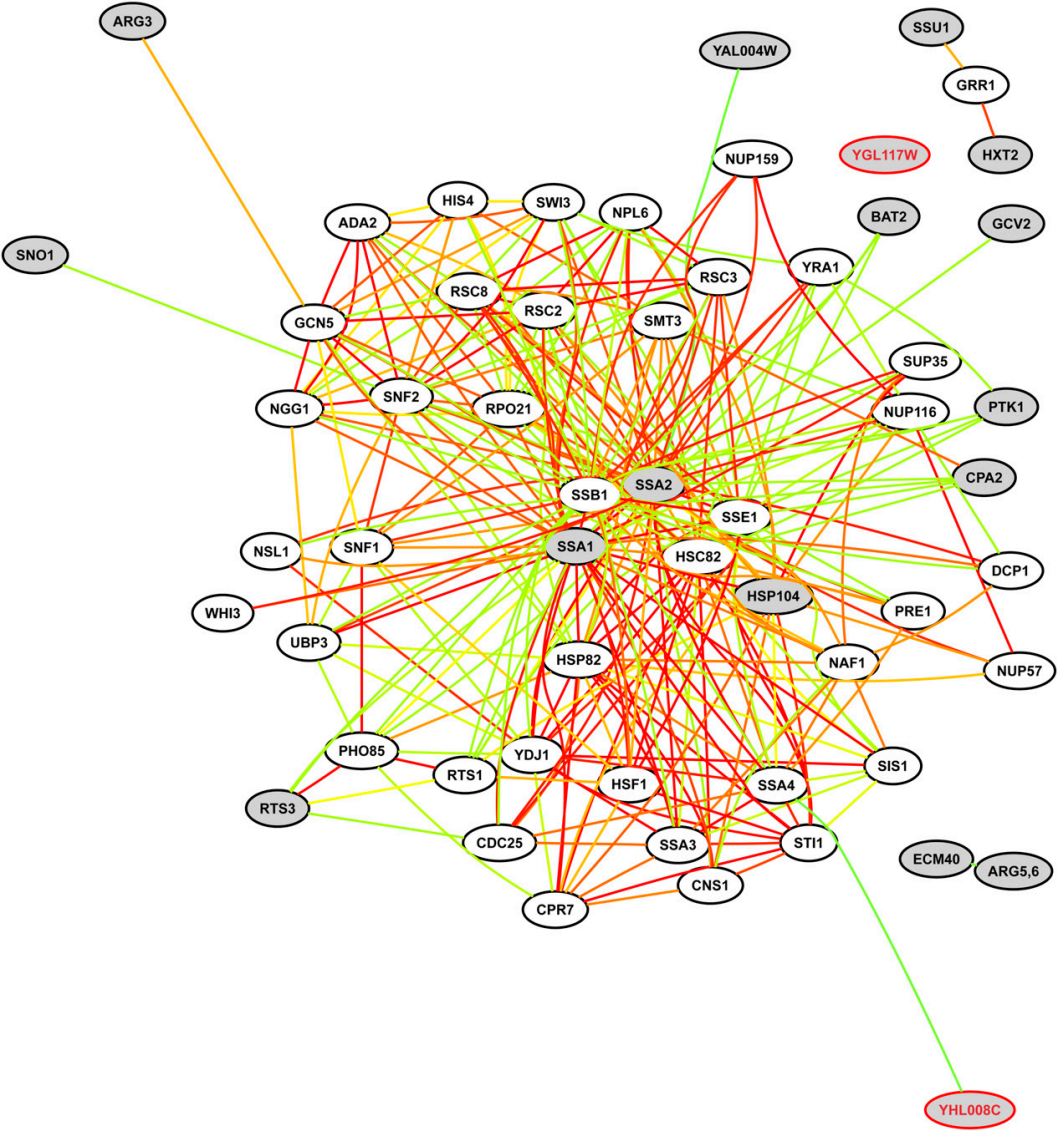
Pathway analysis of coordinated upregulation without Blm10. Gray nodes are genes and ORFs upregulated fourfold to eightfold in *blm10Δ*/*blm10Δ* mutant cells relative to *BLM10/BLM10* cells. YAL004W is a dubious ORF, and YGL117W and YHL008C are uncharacterized ORFs (SGD Project 2010). Confidence-weighted pairwise linkages between genes are color-coded (Myers *et al.* 2005): red (highest confidence), orange, yellow, green (lowest confidence).

We reasoned that mutant cells may compensate by upregulating particular pathways or genes. To evaluate this possibility, we first submitted all genes and open reading frames (ORFs) upregulated greater than fourfold in *blm10Δ*/*blm10Δ* cells (gray nodes in Figure 2) to bioPIXIE (Myers *et al.* 2005) to query whether any of the genes colocalized near each other in a functional network, and if so, what other genes colocalized with them.

The most relevant part of the functional network surrounding the upregulated genes was extracted by bioPIXIE and is shown in Figure 2. Most central and geometrically close in the integrated functional network lie *SSA1*- and *SSA2*-encoded chaperone proteins, homologs of yeast and human members of the heat shock protein 70 multigene family, together with *HSP104* of the Hsp100 family. Ssa1 and Ssa2 are novel G2/M checkpoint proteins that copurify with the DNA damage-dependent checkpoint protein, Rad9, and function after ultraviolet light irradiation in phosphorylating Rad9 and another checkpoint protein, Rad53 (Gilbert *et al.* 2003). It is proposed that the chaperone activities of Ssa1 and Ssa2 remodel the large Rad9 complex to a smaller Rad53 activating complex after genomic insult (Van Den Bosch and Lowndes 2004).

By far, the highest enrichment bioPIXIE measured in the whole network (*P* value 4 × 10^−9^) comprised more than 19% of all genes (Table 5). This specific subset encodes protein-folding chaperones, nine of which were upregulated twofold to sevenfold in mutant cells (Table 6). Those that physically interact with Blm10 (SGD Project 2010) are indicated in Table 6. Eight additional protein-folding genes were enriched (GOLEM, *P* value 5 × 10^−10^) among 144 genes upregulated twofold or more, and are listed in Table 7 with other protein-folding genes regulated ≥1.5 fold. As a group, the encoded molecular chaperones in Figure 2 and Tables 6 and 7 assist to properly fold and assemble nascent polypeptide chains or refold previously denatured or aggregated proteins. Equally important, they interact with partially folded or unfolded protein subunits to stabilize, translocate, or degrade them.

**Table 5:** Gene ontology terms enriched in the gene network shown in Figure 2

Go term	Cluster Frequency	Genome Frequency	*P* Value	Genes
Protein folding	11/57 19.3%	70 / 6471 1.1%	4.10^−9^	*SSA4*, *STI1*, *SSA2*, *SSE1*, *SIS1*, *HSC82*, *HSP82*, *SSA3*, *SSA1*, *HSP104*, *CNS1*

Arginine biosynthesis	4/57 7.0%	10 / 6471 0.2%	3.42^−4^	*ARG3*, *CPA2*, *ARG5*, *6*, *ECM40*

Cellular physiological process	55/57 96.5%	4689/6471 72.5%	7.83^−4^	*SMT3*, *SSA4*, *SSB1*, *HIS4*, *GCV2*, *NUP57*, *NSL1*, *WHI3*, *ARG3*, *STI1*, *HSF1*, *CPA2*, *RTS3*, *RSC8*, *DCP1*, *SSA2*, *SNO1*, *ADA2*, *YDJ1*, *RTS1*, *RSC3*, *SSE1*, *NUP159*, *SUP35*, *YRA1*, *SWI3*, *NPL6*, *NUP116*, *SNF1*, *SIS1*, *GRR1*, *PTK1*, *CDC25*, *PRE1*, *HSC82*, *SSU1*, *PHO85*, *UBP3*, *NGG1*, *HSP82*, *BAT2*, *NAF1*, *RPO21*, *ARG5*, *6*, *SSA3*, *SSA1*, *ECM40*, *SNF2*, *HXT2*, *HSP104*, *YHL008C*, *GCN5*, *TRP5*, *CNS1*, *RSC2*
Cellular process	55/57 96.5%	4728/6471 73.1%	1.19^−3^	*SMT3*, *SSA4*, *SSB1*, *HIS4*, *GCV2*, *NUP57*, *NSL1*, *WHI3*, *ARG3*, *STI1*, *HSF1*, *CPA2*, *RTS3*, *RSC8*, *DCP1*, *SSA2*, *SNO1*, *ADA2*, *YDJ1*, *RTS1*, *RSC3*, *SSE1*, *NUP159*, *SUP35*, *YRA1*, *SWI3*, *NPL6*, *NUP116*, *SNF1*, *SIS1*, *GRR1*, *PTK1*, *CDC25*, *PRE1*, *HSC82*, *SSU1*, *PHO85*, *UBP3*, *NGG1*, *HSP82*, *BAT2*, *NAF1*, *RPO21*, *ARG5*, *6*, *SSA3*, *SSA1*, *ECM40*, *SNF2*, *HXT2*, *HSP104*, *YHL008C*, *GCN5*, *TRP5*, *CNS1*, *RSC2*

**Table 6:** Regulation of protein-folding molecular chaperones encoded by genes in Figure 2

Gene	Fold Change	Encoded Chaperone Activity	Human Homolog or Domain
*SSA2*	↑7.1	Hsp70 family member, member of Rad9 DNA-checkpoint complex	*Hsp70*
*SSA1**^a^*	↑4.2	Hsp70 family, chaperone complex with ADJ1, protein refolding, member of Rad9 DNA-checkpoint complex	*Hsp70*
*HSP104**^a^*	↑4.0	Hsp100 family, acts in conjunction with Ssa1 and Ydj1 (Hsp40), protein refolding	
*SSA3*	↑3.8	Hsp70 family member	*HSPA1AB*
*HSP82*	↑3.3	Hsp90 isoform, associates with Cpr6, Sti1, Cns1, Hch1, Aha1, Sse1, nascent chain folding, protein refolding, proteasome assembly	*HSP90AB1*
*SIS1*	↑2.7	HSP40 (DNAJ) co-chaperone, interacts with Ssa1	*DNAJB1* [*HSP40*]
*SSE1*	↑2.6	Hsp70 family member, component of Hsp90 chaperone complex, protein refolding	*HSPA4*
*HSC82**^a^*	↑2.3	Hsp90 isoform, associates with Sti1, Cns1, Cpr6, Hch1, Aha1, Sse1, nascent chain folding, protein refolding, proteasome assembly	*HS90AB1*
*STI1*	↑1.9	Hsp90 co-chaperone, interacts with Ssa and Hsp70 chaperones	*STIP1*
*SSA4*	↑1.2	Hsp70 family member	*HSPA8*
*CNS1**^b^*	↓1.7	Hsp90 co-chaperone, binds Hsp82 and Ssa1	*TTC4*

a Chaperone physically interacts with Blm10.

b Essential gene.

**Table 7:** Additional protein-folding genes regulated ≥1.5-fold

Gene	Fold Change	Encoded Chaperone Activity	Human Homolog or Domain
*HSP78*	↑3.7	Hsp100 family, mitochondrial homolog of Hsp104, protein refolding	*Clp/Hsp100*
*HSP26*	↑2.9	Small molecular chaperone	
*AHA1*	↑2.6	Co-chaperone, binds Hsp82, activates Hsp90, similar to Hch1	*AHSA1*
*HCH1*	↑2.6	Co-chaperone, binds and activates Hsp90	
*APJ1*	↑2.4	HSP40 (DNAJ) family, regulates Hsp70 activity, genetically interacts with Ydj1	Contains a DNAJ domain
*HSP42*	↑2.3	Small molecular chaperone	
*CPR6*	↑2.3	Binds Hsp82, protein refolding	*PPID*
*CIN1*	↑2.1	Tubulin-folding factor	*TBCD*
*HSP31*	↑1.9	DJ-1/Pfpl family, amino acid substitution in DJ-1 associated with early-onset Parkinson's	*DJ-1*
*YDJ1*	↑1.8	Hsp40 (DNAJ), Ssa1 co-chaperone, regulates Hsp70 and Hsp90 functions, nascent chain folding, protein refolding	*DNAJA2* (*DNAJ* [*Hsp40*])
*HSP60*	↑1.6	Mitochondrial chaperonin, nascent chain folding, protein refolding	*HSPD1*
*HSP10*	↑1.5	Hsp60 co-chaperonin, protein refolding	*HSPE1*
*MPD1*	↓1.9	Endoplasmic reticulum chaperone for glycoproteins	
*SBA1*	↓1.5	Co-chaperone, binds to and regulates Hsp90 family, regulates telomerase activity	p23

Essential genes are underlined.

The Rpn4 transcription factor that stimulates expression of proteasomal genes, positively regulates DNA repair, and physically interacts with Blm10 (SGD Project 2010), was upregulated nearly twofold in mutant cells in the current studies (Table S1). However, the expression of genes encoding components of the 19S and 20S proteasome subunits with which Blm10 physically associates (*e.g.* Pre1-Pre10, Pup2, Pup3, Rpt6, and Scl12; SGD Project 2010) were not significantly altered in cells lacking Blm10, with the exception of *PRE6* and *PUP2*, which were downregulated 31% and 23%, respectively, and *PUP3* which was upregulated 28% (Table S1).

By analogy to the downregulated genes, all of the upregulated genes in these groups may not lead to upregulated protein expression following posttranscriptional and posttranslational modification, or result directly from deleting *BLM10*. Nevertheless, the concurrent upregulated expression of this specific subset of genes produces a chaperone-mediated, protein-folding signature.

In Figure 2, *UBP3/BLM3* is tightly connected and geometrically proximal to *SSA1* and *SSA2* with high confidence (1.0 and 0.9, respectively). Also proximal to these chaperone proteins is *SNF2* (0.65 confidence), encoding the catalytic subunit of the SWI/SNF chromatin remodeling complex involved in nucleosome modification, transcriptional regulation, and DNA double-strand break repair. *NGG1* is also proximal (0.62 confidence), and is involved in chromatin modification as a constituent of histone acetyltransferase complexes.

### Proteolysis

Having discovered *UBP3/BLM3* is linked with the highest confidence to both highly upregulated genes most central in the network of upregulated genes in *blm10Δ*/*blm10Δ* cells (Figure 2), we compared the degradation of β-galactosidase in *blm3-1* cells with that in wild-type and *blm10Δ* cells. Cells were transformed with pUB23, a plasmid containing an ubiquitin-*lacZ* gene fusion under the control of a GAL promoter that allows gene expression to be controlled by the amount of galactose supplied in media (Bachmair *et al.* 1986). After induction of the fusion protein, ubiquitinated β-galactosidase is produced, and the ubiquitin tag automatically targets β-galactosidase for degradation by proteasomes (Bachmair *et al.* 1986).

**Figure 3:**
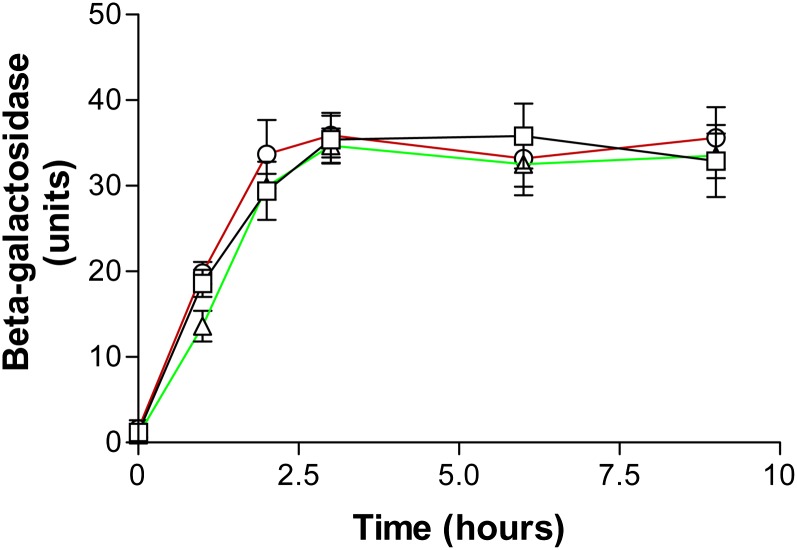
Degradation of β-galactosidase by proteasomes in normal (□), *blm3-1* (Ο), and *blm10Δ* (Δ) strains. Enzymatic activities in cells were determined spectrophotometrically at the indicated time points.

Time-dependent β-galactosidase activities in the three strains are shown in Figure 3. Activities were barely detectable at time zero but increased after the first hour and for two additional hours before reaching a plateau. Comparable activities of the three genotypes indicated Ubp3/Blm3 and Blm10 do not stimulate hydrolysis of this model and intact, folded protein. Our Blm10 results are consistent with structural restraints of the small dome-like opening of Blm10/PA200-proteasome complexes through which small peptides, but not proteins, might pass (recently reviewed in Savulescu and Glickman 2011; Stadtmueller and Hill 2011; Dange *et al.* 2011). They also are consistent with previous reports that Blm10 appears not to contribute significantly to the degradation of other ubiquitinated proteasome substrates (Schmidt *et al.* 2005a), and that Blm10/PA200 binding to the core particle activates it for hydrolysis of some peptides (Ustrell *et al.* 2002; Schmidt *et al.* 2005a; Iwanczyk *et al.* 2006; Lehmann *et al.* 2008; Lopez *et al.* 2011; Dange *et al.* 2011). The specific peptide substrate affects the amount of hydrolysis by Blm10/PA200 *in vitro* (Ustrell *et al.* 2002; Fehlker *et al.* 2003; Lopez *et al.* 2011; Dange *et al.* 2011).

### Global DNA damage is conferred by the *blm10Δ* and *blm3-1* mutations

As a logical follow-up to the comparative genome analyses, we evaluated the genomic integrity in diploid cells without Blm10 and included *blm3-1/ blm3-1* diploid cells in these studies. For these chromosomal studies, we used pulsed-field gel electrophoresis, during which individual chromosomes separate into distinct bands according to molecular weight and electric field interaction, and double-strand breaks in DNAs cause bands to lessen in intensity or disappear in a dose-dependent manner (Moore *et al.* 2000). Degraded chromosomes either leave the gel or accumulate at the bottom as a diffuse smear. The bleomycin-phleomycin family of chemical congeners is used as a tool in DNA damage studies (*e.g.* Moore 1990, 1999; Moore *et al.* 2000), and we used phleomycin to examine the quality of chromosomal DNAs in wild-type diploid cells (*BLM10/BLM10*, *BLM3/BLM3*) and mutant diploid cells in which either *BLM10* was deleted (*blm10Δ*/*blm10Δ*, *BLM3/BLM3*) or *UBP3/BLM3* was truncated (*BLM10/BLM10*, *blm3-1lblm3-1*) on homologous chromosomes.

**Figure 4:**
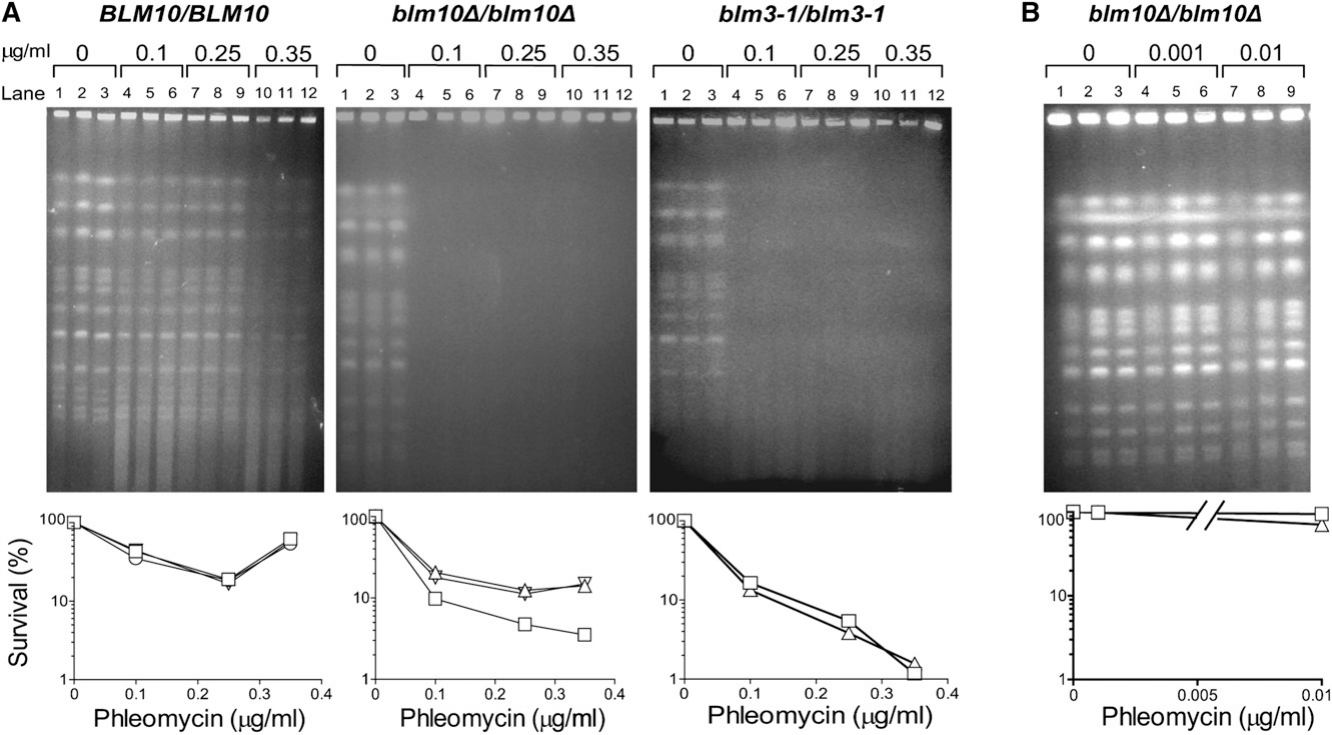
Pulsed-field gel electrophoretic analyses comparing chromosomal damage and killing after no treatments and after 30-min phleomycin treatments. Diploid genotypes with respect to *BLM10* and *BLM3* were *BLM10/BLM10*, *BLM3/BLM3*; *blm10Δ/blm10Δ*, *BLM3/BLM3*; and *BLM10/BLM10*, *blm3-1/blm3-1*. Treated populations were divided and incubated under nongrowing conditions for 24 or 48 hr, during which competent strains can reconstruct their chromosomes (Moore *et al.* 2000). Routine microscopic examination and visual counting of cells before and after these LH periods confirmed cell populations did not bud or grow, and cell lysis was never observed. (A) Lanes 1, 4, 7, and 10, no LH. Lanes 2, 5, 8, and 11, 24-hr LH. Lanes 3, 6, 9 and 12, 48-hr LH. (B) Lanes 1, 4, 7, no LH. Lanes 2, 5, 8, 24-hr LH. Lanes 3, 6, 9, 48-hr LH. Corresponding survival data: squares, 0 LH; inverted triangles, 24-hr LH; triangles, 48-hr LH. Pulsed-field gel electrophoreses and survival analyses are representative of three independent experiments and of multiple diploid constructions of the same genotypes.

Without treatment, chromosomes from all three diploids consistently produced strong bands (Figure 4, A and B, lanes 1−3). In wild-type cells, chromosomal bands remained relatively strong after 0.1 μg/mL (lanes 4−6; Figure 4A) and 0.25 μg/mL (lanes 7−9; Figure 4A) phleomycin treatments, and underwent moderate degradation after 0.35 μg/mL treatments (lanes 10−12; Figure 4A). However, chromosomes in *blm10Δ*/*blm10Δ* and *blm3-1/blm3-1* cells degraded following the same three treatments (lanes 4−12 *vs.* lanes 1−3; Figure 4A), indicating marked deficiencies. Exposure to 10- to 100-fold lower doses left chromosomes in *blm10Δ/blm10Δ* cells intact (Figure 4B).

Cell death, measured in parallel with DNA damage in wild-type, *blm10Δ/blm10Δ*, and *blm3-1/blm3-1* cells in each experiment, was low in wild-type cells and increased after 0.1 and 0.25 µg/mL phleomycin treatments (Figure 4A). Cell death was greater in mutant cells than in wild-type cells (Figure 4, A and B), indicating protective functions of Blm10 and Ubp3/Blm3. The order of decreasing resistance among the diploids was *BLM10/BLM10*, *BLM3/BLM3* > *blm10Δ*/*blm10Δ*, *BLM3/BLM3* > *BLM10/BLM10*, *blm3-1lblm3-1* > *blm10Δ*/*blm10Δ*, *blm3-1lblm3-1*.

Consistently, we observed lower cell death in wild-type cells after 0.35 µg/mL treatments than after 0.25 µg/mL treatments, but not in mutant cells (Figure 4A). The reduced cell death in wild-type cells is most likely attributable to induced DNA repair and cellular recovery observed after this drug family causes extensive chromosomal damage (Moore *et al.* 2000).

### Susceptibilities to lethal effects of agents with different mechanisms of action

The comparative genome analyses together with finding elevated DNA damage and killing after phleomycin treatments led us to examine whether the absence of Blm10 heightened susceptibilities to agents with different mechanisms of action. For these studies, we selected agents we previously had not investigated.

**Figure 5:**
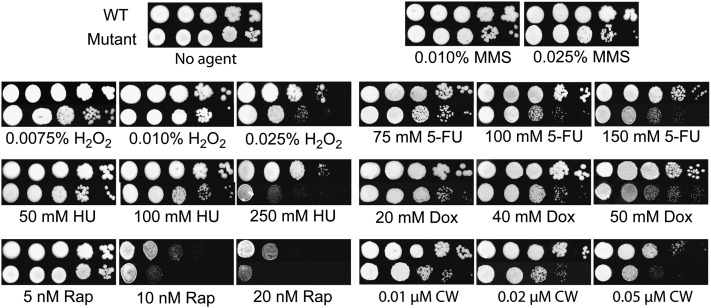
Dose-dependent susceptibilities of normal and mutant diploids. From left to right are fivefold serial dilutions of each genotype. MMS indicates methyl methanesulfonate; H_2_O_2_, hydrogen peroxide; 5-FU, 5-fluorouracil; HU, hydroxyurea; Dox, doxorubicin; Rap, rapamycin; CW, calcofluor white. Results are representative of two to four independent experiments.

As shown in Figure 5, Blm10 loss reduced resistance to low concentrations of methyl methanesulfonate and strikingly compromised defenses against low-dose treatments of hydrogen peroxide, 5-fluorouracil, hydroxyurea, and doxorubicin. Considered oxidant mutagens, the bleomycin-phleomycin family and hydrogen peroxide cause similar DNA damage (Demple and Harrison 1994). Some of the gene functions important for resistance to these agents (*e.g.* Moore 1978, 1982; Moore *et al.* 1992, 2000; Aouida *et al.* 2004; Cloos *et al.* 2006) overlap with those required for resistance to ionizing radiation (Bennett *et al.* 2001) and some other chemicals (Parsons *et al.* 2004). In contrast, anticancer 5-fluorouracil and hydroxyurea are antimetabolites (Brunton *et al.* 2006; Friedberg *et al.* 2006; Nitiss and Heitman 2007). 5-fluorouracil is a pyrimidine antagonist that is synthesized into 5-fluoro-2-deoxyuridine, a nucleotide that inhibits the 2-deoxythymidine synthesis by thymidine synthetase. Incorporation of 5-fluorouracil into DNA interferes with DNA synthesis and inhibits RNA production. Hydroxyurea inhibits DNA and RNA synthesis by blocking ribonucleotide reductase and causes site-specific DNA damage through the formation of hydrogen peroxide and nitric oxide (Sakano *et al.* 2001). The anthracycline doxorubicin intercalates between DNA bases, causing DNA breaks by blocking topoisomerase type II (Brunton *et al.* 2006). Moreover, DNA can be damaged by oxidative free radicals generated during the metabolism of doxorubicin (Turner *et al.* 1990).

Mutant cells also lost protection against rapamycin (Figure 5), a novel anticancer antibiotic first identified as antifungal (Brunton *et al.* 2006; Nitiss and Heitman 2007). Immunosuppressant rapamycin arrests cells at G1/S and inhibits highly conserved nutrient sensing pathways. The elevated sensitivity of the mutant cells to rapamycin perhaps could be explained by the knowledge that this drug strongly induces *BLM10* (Hardwick *et al.* 1999) and increases *BLM10* mRNA levels (Lopez *et al.* 2011), and these events would not be possible in *blm10*-deleted cells.

Finally, bleomycin damages fungal cell walls (Moore *et al.* 1992; Beaudouin *et al.* 1993; Moore *et al.* 2003), as does cell-wall perturbing calcofluor which binds chitin, thereby inhibiting chitin synthase and proper cell wall biosynthesis (Nitiss and Heitman 2007). As Figure 5 shows, cells lacking Blm10 were hypersusceptible to calcofluor. It is tempting to suggest that this sensitivity could be attributable to the downregulation of *MEC3*, cell-cycle checkpoints, transcription factors, and cytokinesis in these cells (Table 4). As a whole, the results shown in Figure 5 suggest inhibition by Blm10/PA200 could dually target cancer and fungal cells.

In similar experiments, normal and mutant strains grew equally well after mitomycin C, ultraviolet light irradiation, and ethidium bromide treatments (data not shown). This could reveal discernment of the type of DNA damage because the modes of action of these agents differ from those in Figure 5. Mitomycin C is a potent DNA cross-linker (Friedberg *et al.* 2006; Deans and West 2011). Ultraviolet radiation produces cyclobutane pyrimidine dimers in DNAs, along with pyrimidine-pyrimidone (6-4) and other photoproducts (Friedberg *et al.* 2006). Ethidium bromide intercalates in DNA (Reinhardt and Krugh 1978), but differs from doxorubicin in its action.

In a previous study, *BLM10* and *blm10Δ* strains were reported to grow equally after treatments with gamma irradiation, methyl methanesulfonate, ultraviolet irradiation, and camptothecin (Iwanczyk *et al.* 2006). In the same study these strains also grew equally after treatments with bleomycin, phleomycin, and hydroxyurea (Iwanczyk *et al.* 2006), in contrast to the hypersusceptibilities *blm10Δ* cells exhibited to these drugs in the current studies (Figures 4 and 5) and to the bleomycin-phleomycin family in previous studies (Febres *et al.* 2001; Doherty *et al.* 2004; Schmidt *et al.* 2005a).

### Growth of *BLM10/BLM10* and *blm10Δ*/*blm10Δ* strains at 37°

Previously, a temperature-sensitive phenotype was reported for *blm10Δ* cells growing at 37° in one strain background (Fehlker *et al.* 2003) but not in others (Schmidt *et al.* 2005a; Iwanczyk *et al.* 2006). We compared the growth of *BLM10/BLM10* and *blm10Δ*/*blm10Δ* strains in several experiments in the current studies and found that they grew equally well at 37°. This finding was confirmed in quantitative growth measurements by hemacytometer counts of cells from 0 to 72 hr in three independent experiments (K. Doherty and J. Lukose, unpublished data).

### Respiratory deficiency

The functional state of mitochondria affects resistance to killing by the bleomycin-phleomycin family of DNA-cleaving drugs, as respiratory-deficient cells lacking mitochondrial DNA (ρ^0^) are up to 100-fold more resistant than isogenic respiratory-proficient (ρ^+^) cells (Davermann *et al.* 2002). During the current studies, we found that *blm10Δ* and *blm10Δ/blm10Δ* strains growing in the absence of bleomycin or phleomycin produced up to tenfold higher frequencies of respiratory-deficient petite colonies than *BLM10* and *BLM10/BLM10* strains (L. Pride, K. Doherty, and C.W. Moore, unpublished data). Other *blm10Δ* haploid strain backgrounds also produced increased yields of petite colonies (Sadre-Bazzaz *et al.* 2010).

### Blm10 localization during the cell cycle

The cellular localization of the YFP-Blm10 fusion protein was determined in live cells by fluorescence microscopy. YFP-Blm10 was the sole copy of Blm10 in the cell and gene expression was driven from its endogenous promoter in its native chromosomal context. The protein is completely functional in totally relieving phleomycin hypersensitivity in *blm10Δ* and *blm10Δ*/*blm10Δ* mutant cells.

**Figure 6:**
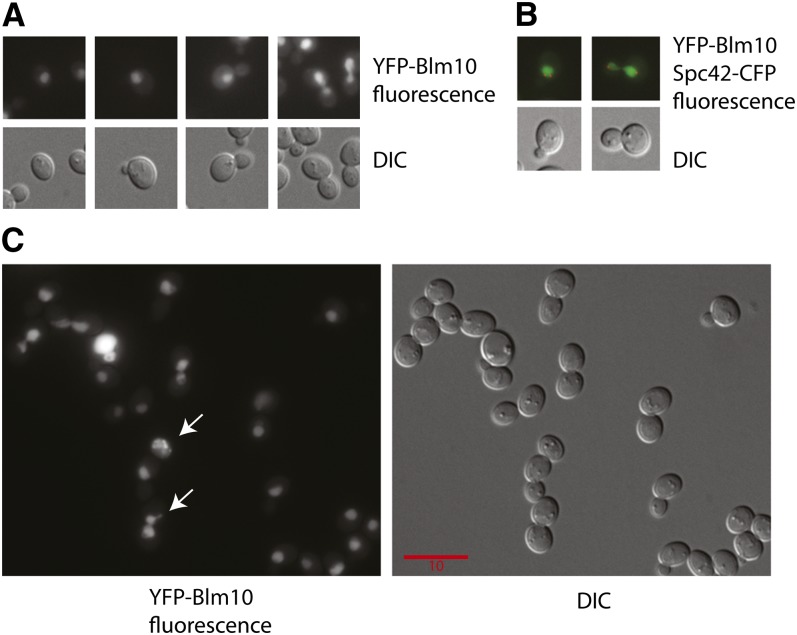
Nuclear localization and colocalization of Blm10. Shown are representative YFP and differential interference contrast (DIC) images of living cells. The scale bar applies for all images in the panels as all images are scaled equally. Representative cells from populations of 4000 to 10,000 cells are shown to illustrate the following: (A) Nuclear localization, showing progression from unbudded to large-budded cells. (B) Colocalization of nuclear Spc42-CFP and YFP-Blm10. (C) Nuclear localization after growth on medium supplemented with 20 μg/mL phleomycin D_1_. Arrows indicate cells with evidence for nuclear membrane fragmentation.

YFP-Blm10 localized to nuclei in all budded and unbudded cells (Figure 6A), consistent with our findings that Blm10 protects against DNA damage, and that the yeast 26S proteasome complex is predominantly nuclear (80%; Russell *et al.* 1999). The identification of the nucleus was confirmed by coexpressing YFP-Blm10 with CFP-tagged Spc42 (Figure 6B), a component of the spindle pole body that is embedded in the nuclear membrane (Muller *et al.* 2005).

The levels of Blm10 in the nucleus were measured quantitatively as described in *Materials and Methods* and found not to be constant during the cell cycle. The fluorescence intensity of YFP-Blm10 in budded cells, before nuclear migration to the neck, was approximately 40% greater than in unbudded G1 cells. Thus, after DNA replication Blm10 becomes enriched in the nucleus during late S and early M phases, before anaphase and cell division. The enrichment at the S/M transition of the cell cycle is when the state of the DNA is monitored by checkpoint controls.

### Blm10 localization signal and pattern after DNA damage

We compared the localization signals and patterns after DNA damage to those before DNA damage. Cells were treated with 20 µg/mL phleomycin D_1_ for 4 hr. After this treatment, fluorescence was relatively uniform throughout the nucleus. A heterogeneous response was observed, with some cells showing a large increase in the level of YFP-Blm10 in the nucleus and some cells showing evidence of nuclear fragmentation (Figure 6C).

### Identification of conserved regions in different parts of the Blm10 protein

The Blm10 protein is highly conserved among diverse organisms, from yeast to humans. Blm10/PA200 is the most conserved proteasome activator (Finley 2009). In addition, all homologs are quite large, with 240-kD Blm10 being the largest (http://blast.ncbi.nlm.nih.gov/Blast.cgi).

**Figure 7:**
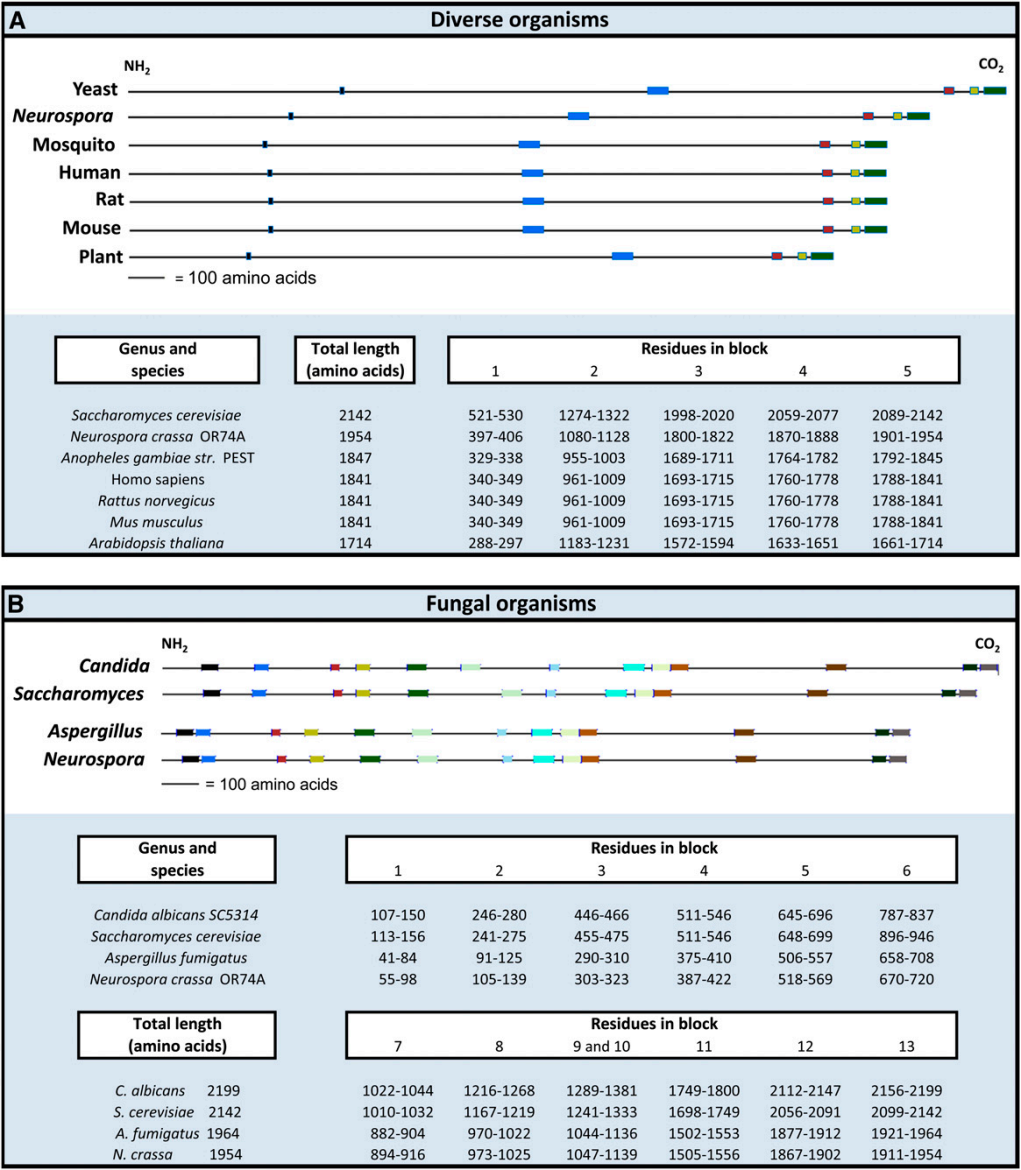
Evolutionarily conserved sequences among Blm10 homologs. The sequences are kept to scale to show the relative location and spatial arrangements of the conserved regions. Homologs were aligned by the global multiple sequence alignment program, CLUSTALW (http://www.ddbj.nig.ac.jp/search/clustalw-j.html). Proteins are arranged in order of decreasing size in (A) and (B). (A) Dissimilar distances among some of the homologs between the N-terminus and first conserved block, first and second block, and second and third block contrast with the relatively similar distances between carboxyl-conserved regions in all homologs. (B) Similarities among conserved regions as described in the text.

We arbitrarily selected seven of these homologs and used the Blocks database (Henikoff *et al.* 2000) to multiply align ungapped Blm10 regions to them. This identified five conserved regions (Figure 7A). Comparing the yeast sequence to the other sequences, we found that the positions of the first two conserved regions, and the distances between these two conserved regions, varied most (Figure 7A). In contrast, the positions and spacing of the three carboxyl-terminal regions were more conserved among all seven sequences. The sizes of the human, rat, and mouse proteins and the positions of all five conserved sequences in those proteins were identical (Figure 7A). The mosquito sequence was most similar to these three in size and locations of conservation.

The Blm10 sequences of two molds and two fungi that grow as yeasts also were arbitrarily selected and compared (Figure 7B). Interestingly, the sizes and locations of the conserved regions of the *Neurospora crassa* homolog were most similar to those in the other mold, *Aspergillus fumigatus*. By comparison, the sizes and locations of conserved regions of Blm10 in *S. cerevisiae* were most similar to those in *Candida albicans*.

### Protection does not require the largest conserved region at the carboxyl-terminus

**Figure 8:**
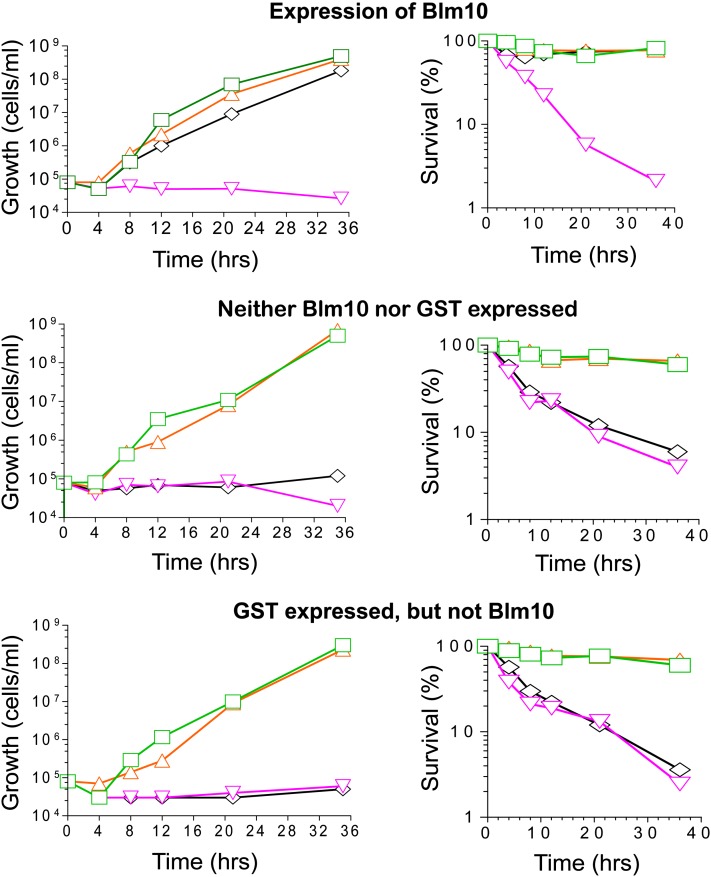
Resistance conferred by truncated Blm10. Expression of Blm10_(-339aa)_-GST from an inducible pCUP1 promoter was controlled by adjusting the amounts of copper added to media (Mascorro-Gallardo *et al.* 1996). Top: *blm10Δ* cells expressing *BLM10*. Middle: *blm10Δ* cells not expressing *BLM10* or GST. Bottom: *blm10Δ* cells expressing GST but not *BLM10*. Left: cell densities during growth. Right: survival. Green rectangles indicate no copper or phleomycin; orange triangles, 50 μM copper, no phleomycin; inverted pink triangles, 5 μg/mL phleomycin, no copper; black diamonds, 50 μM copper, 5 μg/mL phleomycin.

We investigated activities of a truncated Blm10 protein (Figure 1) lacking the three carboxyl-terminal conserved regions (blocks 3, 4, and 5 in Figure 7A). Without drug, mutant cells grew normally whether or not Blm10_(-339aa)_ was induced (Figure 8). However, Blm10_(-339aa)_ rescued growth-inhibition and cell death in the presence of 0.5, 1, 3, 5, 7, and 9 µg/mL phleomycin. For example, as shown in Figure 8, *blm10Δ* conferred complete inhibition of growth in the presence of 5 µg/mL phleomycin, and survival of the treated cells decreased to 2% by 35 hr unless Blm10_(-339aa)_ was produced (Figure 8, top row). These experiments demonstrated Blm10_(-339aa)_ was functional when produced as a GST-fusion, and its expression rescued growth inhibition and cell death. Similarly, the largest conserved region was not required for relief of phleomycin hypersusceptibility in *blm3-1* mutant strains (Febres *et al.* 2001; Doherty *et al.* 2004). In experiments not shown here, overexpression of Blm10_(-339aa)_ did not produce additional phenotypes.

Control experiments confirmed Blm10_(-339aa)_ expression was required to rescue growth inhibition and cell death because mutant cells without plasmid-expressed Blm10_(-339aa)_ were growth inhibited and survived poorly (Figure 8, middle and bottom rows). Copper had no effect on growth or killing because each was comparable with and without copper.

Of note, haploid and homozygous diploid strains encoding Blm10_(-339aa)_ under the control of its endogenous promoter exhibit higher sensitivities to killing by phleomycin than strains of the same ploidy with the entire gene deleted, and their sensitivities were nearly as high as *blm3-1* strains (Febres *et al.* 2001; Doherty *et al.* 2004). In a different strain background, a truncated deletion also caused higher bleomycin sensitivity than the full deletion (Schmidt *et al.* 2005a). Heterozygous strains bearing the truncated *BLM10* allele exhibited intermediate resistance (Febres *et al.* 2001; D. Febres and K. Doherty, unpublished data).

### Localization of Blm10_(-339aa)_

**Figure 9:**
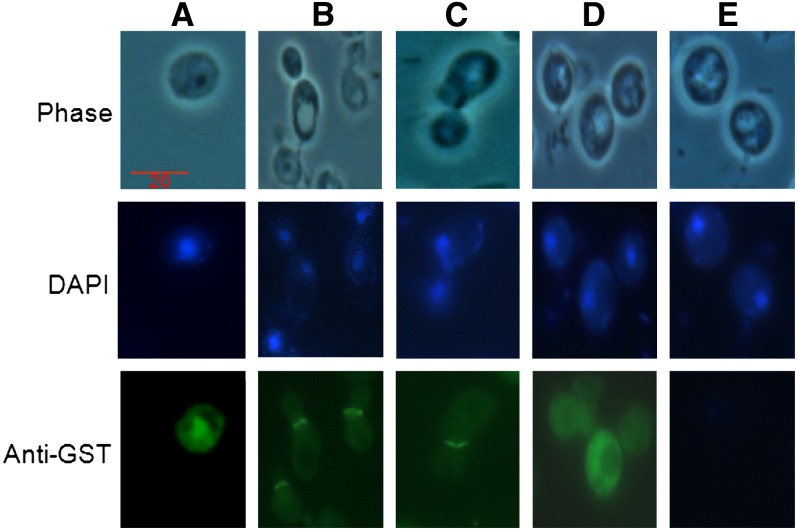
Blm10_(-339aa)_ localization. The truncated Blm10 protein was induced with 50 μM copper because this concentration produced a stable protein and functionally relieved drug hypersensitivity. The representative wild-type cells illustrate two populations of cells, one unbudded (A) and the other budded (B and C). The nuclear localization is maintained in unbudded cells, in contrast to the bud-neck localization in budded cells. Top row: phase contrast. Middle row: DAPI staining of DNA. Bottom row: cells after GST antibody staining. Column A: unbudded cells. Columns B and C: different populations of budded cells. Column D: cells expressing GST but not Blm10. Column E: no antibody treatment. Because of the truncated protein, these cells are somewhat distended. In addition, before treatments with DAPI and anti-GST antibody, cells are converted to spheroplasts, which causes distortion of cells that have lost their cell wall integrity.

Using the same Blm10_(-339aa)_-GST, we investigated its localization and whether the conserved C-terminus was required for nuclear localization. Immunofluorescence microscopy localized Blm10_(-339aa)_-GST with GST antibodies, and various copper concentrations controlled protein amounts. Like full-length Blm10, Blm10_(-339aa)_ localized in nuclei of stationary phase (G0) cells, as confirmed by DAPI staining of DNA (*e.g.*, Figure 9A). During G1, S, G2, and M phases, the fluorescence formed a disk at the bud neck or septum between mother and daughter cells (*e.g.* Figure 9, B and C). It appeared to arch and follow the contour of the mother cell rather than the daughter (*e.g.*, Figure 9C). Expressing GST alone revealed diffuse cytoplasmic staining (Figure 9D), eliminating the GST as the cause for the localization patterns. Without staining, no significant autofluorescence was observed (Figure 9E).

The localization we observed in budded cells and the cytoplasmic distribution of Blm10 observed previously (Schmidt *et al.* 2005a) suggest that a nuclear localization sequence could be encoded in the truncated region. Database searches identified a strong, although not canonical, nuclear localization sequence in that region (Schmidt *et al.* 2005a).

## Discussion

Cells have multiple protein complexes to keep the genome intact and functional. Based on the current studies, the relationship among the components of the complexes may well differ between cells with and without the Blm10-20S proteasome activator. Loss of the activator downregulated numerous genes crucial for maintaining genomic stability, heightened DNA damage, and selectively sensitized cells to agents with different mechanisms of action. Although the exact or direct cause of the elevated DNA damage in Blm10 cells was not investigated for the current studies, the simultaneous downregulated expression appears important and suggests ways that Blm10 loss predisposes cells to lethal effects of agents with diverse mechanisms of action. We propose that modulated chromatin structure could compromise DNA integrity in *blm10Δ/blm10Δ* mutant cells, and conclude that the hypersusceptibilities establish Blm10 as a guardian against cellular stresses.

Even without external stimuli, cells were stressed without Blm10 because components of the chaperone and co-chaperone Hsp90 complex, Hsp70 subfamily, and Hsp100 family were selectively upregulated. This upregulation took place despite the fact that the chemostat regime guaranteed the growth rate remained the same in wild-type and mutant cultures. Consistent with its protection of chromosomes, Blm10 remained in nuclei throughout the cell cycle. The findings contribute knowledge that provides a strong foundation for future studies of the role(s) of Blm10 in relationship to functional groups of proteins, and of the mechanisms of the downregulated and upregulated genes, pathways and signaling networks.

The results also have implications for translational studies of ubiquitin-independent targeting in anticancer therapy. PA200/Blm10 inhibition could be a novel approach to cancer treatment, either alone or in combination with targeted inhibition of one or more of the upregulated chaperones. In fact, cytokinesis was the most enriched process among genes downregulated 25% or more in the current study, suggesting that inhibiting Blm10/PA200 could cause cancer cells to fail to divide. That proteasome inhibitors upregulate molecular chaperones in mammalian cells (Wyttenbach *et al.* 2000; Mitsiades *et al.* 2002; Pritts *et al.* 2002; Awasthi and Wagner 2005; Zaarur *et al.* 2006) supports our predictions. However, control experiments such as the steady-state conditions in the current report were not possible in mammalian cells to determine whether the general mammalian stress response was induced.

Blm10 physically associates with proteins that can assist in mediating protection and maintaining the integrity of chromosomal DNA (SGD Project 2010). Three of these proteins, Sir4, Zds2, and Dun1, are involved in maintaining chromosomal integrity and promoting DNA repair. Genetic interaction between Blm10 and the Dun1 DNA damage checkpoint kinase causes a synthetic growth defect (Pan *et al.* 2006). Without Blm10, *DUN1* was downregulated 50% in the current studies, *ZDS2* was upregulated 10%, and the expression of *SIR4* was not significantly changed (Table S1). Sir4 and Zds2 are important for efficient DNA repair by nonhomologous end-joining (Lewis and Resnick 2000), the major DNA repair pathway in human cells. Sir4 leaves telomeres after DNA damage and relocates to double-strand breaks, where it binds the well-established component of double-strand break repair, yKu70 (Martin *et al.* 1999). Zds2 confers resistance to the anticancer DNA damaging drug cisplatin (Burger *et al.* 2000), suppresses defects resulting from histone mutations (Ma *et al.* 1996), and suppresses mutations in the *CDC20* gene required for chromosome segregation (Yu *et al.* 1996).

Interacting proteins also include the molecular chaperones, Hsp104, Hsc82, Ssz1, Ump1, and Zuo1. When Blm10 was absent, *HSP104* and *HSC82* were upregulated fourfold and 2.3-fold, respectively (Table 6). *SSZ1* and *UMP1* were upregulated 30% and 10%, respectively (Table S1).

Blm10 and Ubp3/Blm3 physically associate or exhibit genetic interactions with four of the same proteins, Hsc82, Sir4, Rpn4, and Ump1, a short-lived chaperone involved in ubiquitin-mediated proteolysis and protein folding (SGD Project 2010). These shared interactions may have facilitated compensation for the *blm3-1* mutant defect by Blm10 overexpression (Febres *et al.* 2001; Doherty *et al.* 2004) and could link the two proteins. It is unknown why the *blm3-1*mutant phenotype was not suppressed by Blm10 overexpression from a different construct in a later study (McCullock *et al.* 2006).

Based upon all of the results presented in the current report, and strengthened by the functional knowledge of proteins with which Blm10 physically and genetically interact, we propose that Blm10 acts to mediate DNA damage and other stresses. For example, 20S proteasomes effectively degrade and remove oxidatively damaged histones and other proteins. Chromatin reorganization would then allow for efficient DNA repair. We further propose that the activated Blm10-CP complex may be involved in removing toxic agents or detoxifying them. In future studies, it would be informative to determine which of the downregulated genes contribute to the vulnerability of *blm10Δ* cells to agents with different mechanisms of action. It is possible that at least some of the downregulated genes may cause the slower growth of *blm10Δ/blm10Δ* mutant diploids than wild-type diploids that we observe in and on limited or synthetic media outside of chemostats.

We also propose a role for Ubp3/Blm3 in protection based on its central position and connection to *SSA1* and *SSA2* in the upregulated protein network (Figure 2) and the excessive chromosomal damage and lethality in *blm3-1/blm3-1* cells (Figure 4). *UBP3* was isolated as a multicopy suppressor of the temperature sensitivity of *S. cerevisiae* cells doubly mutant for the *SSA1* and *SSA2* molecular chaperone genes (Baxter and Craig 1998). Interestingly, these two chaperone-encoding genes were the most highly upregulated in *blm10Δ*/*blm10Δ* cells in the current studies (Table 6). In addition, it is known that Ubp3 binds Sir4 and regulates chromosomal silencing, possibly by controlling the activity or assembly of the Sir complex (Moazed and Johnson 1996). It has been proposed that by deubiquitinating misfolded proteins, Ubp3 permits protein refolding, stability and function (Brew and Huffaker 2002). The *S. cerevisiae* Rad4 protein binds ultraviolet light-damaged DNA and promotes nucleotide excision repair (Mao and Smerdon 2010). Recently, it was suggested that Ubp3 physically interacts with the 26S proteasome and the Rad4 protein to help degrade Rad4 and suppress DNA repair (Mao and Smerdon 2010).

Results in this report indicate Blm10 protects cells from genomic instability and cell death. Consistent with our findings, PA200-knockdown cells showed genomic instability and reduced survival after ionizing irradiation (Blickwedehl *et al.* 2008). We reason that protein interactions and signaling cascades respond to DNA damage to arrest the cell cycle and repair DNA, recruit chromatin remodeling and DNA repair proteins, and recruit chaperones to assist with ridding cells of dysfunctional proteins or toxic agents. Molecular chaperones can repair nonfunctional or misfolded proteins or proteins can be ubiquitinated and targeted to the 26S proteasome for degradation. Neither PA200 nor Blm10 activates the proteasomal 20S catalytic chamber in response to ubiquitinated proteins or requires the activity of ATPase for proteasomal cleavage (Ustrell *et al.* 2002; Schmidt *et al.* 2005a), unlike the ubiquitin-dependent, ATPase-dependent 19S regulatory particle.

Although the protective function of Blm10 does not require its carboxyl-terminal region (Figure 8), it does require the two new conserved regions identified in these studies (Figure 7A). The truncated protein is missing the last three residues (TyrTyrAla) that structural analyses show make very close contact with the 20S proteasome (Sadre-Bazzaz *et al.* 2010). In addition, manipulation of these residues by mutagenesis alters Blm10 function (Lopez *et al.* 2011; Dange *et al.* 2011). Thus, the protective function appears not to require that Blm10 associate with the 20S proteasome, or the truncated Blm10 can associate with the proteasome without this region.

The truncated protein may be missing a domain that disallows its association or interaction with one or more other proteins. The carboxyl-terminus is essential for retention in the nucleus at all stages of growth, since the truncated protein mislocalized in a prior study (Schmidt *et al.* 2005a) and in budded cells in the current studies. It is not known if the septin localization relates to the physical association of Blm10 with a protein involved in bud site selection, Bud20 (Ho *et al.* 2002), which could pull the complex to the septin. This could underlie the increased calcofluor sensitivity of *blm10Δ*/*blm10Δ* cells (Figure 5).

Finally, results presented here suggest a 20s proteasome activator could be a target for proteasome inhibition in combination anticancer therapies. Multiple myeloma cells, for example, have increased proteasome levels and activity (Wada *et al.* 1993; Edwards *et al.* 2009) and circulating proteasomes (Jakob *et al.* 2007). Proteasome inhibition in these cells inhibits proliferation, induces apoptosis, and overcomes drug resistance (Hideshima *et al.* 2001; Edwards *et al.* 2009). In addition to targeting PA200/Blm10 and one or more of the upregulated proteins, targeting it with a conventional treatment could be effective.

## Supplementary Material

Supporting Information
