# Supplementary material for: Loss of a 20S Proteasome Activator in Saccharomyces cerevisiae Downregulates Genes Important for Genomic Integrity, Increases DNA Damage, and Selectively Sensitizes Cells to Agents With Diverse Mechanisms of Action
Source: G3 (Bethesda). 2012 Aug 1;2(8):943–59. doi: 10.1534/g3.112.003376 (PMC3411250; doi:10.1534/g3.112.003376)
Supplement: Supporting Information [file supp_2_8_943__index.html]

Supporting Information 

# Loss of a 20S Proteasome Activator in *Saccharomyces cerevisiae* Downregulates Genes Important for Genomic Integrity, Increases DNA Damage, and Selectively Sensitizes Cells to Agents With Diverse Mechanisms of Action

## Supporting Information for Doherty *et al.*, 2012

**Files in this Data Supplement:**

- Table S1 - Microarray data (.xls 1.1 MB)
